# Supplementary material for: Risk alleles for tuberculosis infection associate with reduced immune reactivity in a wild mammalian host
Source: Proc Biol Sci. 2019 Jul 17;286(1907):20190914. doi: 10.1098/rspb.2019.0914 (PMC6661349; doi:10.1098/rspb.2019.0914)
Supplement: Principal components analysis (PCA) of genetic structure among herds; Cytokine production over time by multi-locus genotype [file rspb20190914supp1.pdf]

<u>Supplementary Material:</u>

Tavalire HF, Hoal EG, le Roex N, van Helden PD, Ezenwa VO, Jolles AE. Risk alleles for tuberculosis infection associate with reduced immune reactivity in a wild mammalian host. *Proceedings of the Royal Society B*. DOI: 10.1098/rspb.2019.0914.

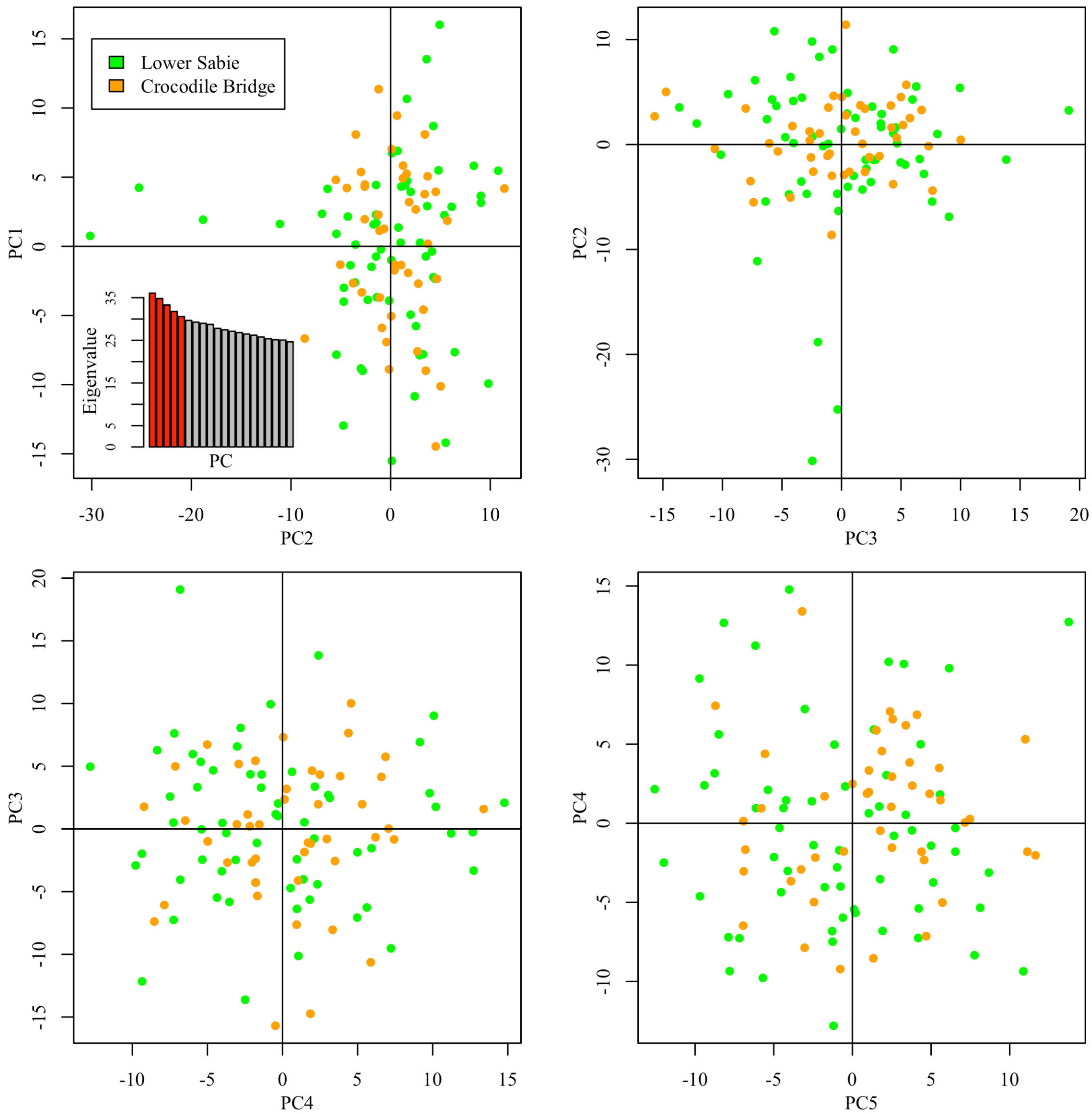

Supplemental Figure S1: Principal components analysis (PCA) of genetic structure among herds. Each panel shows individuals plotted by sequential combinations of the first five principle components, color-coded by sample herd. Here we see no obvious clustering based on herd membership. The eigenvalues of the first twenty principle components are plotted as an inset in the top left panel with the first five PCs highlighted in red. The decrease in eigenvalue levels off after principle component five.

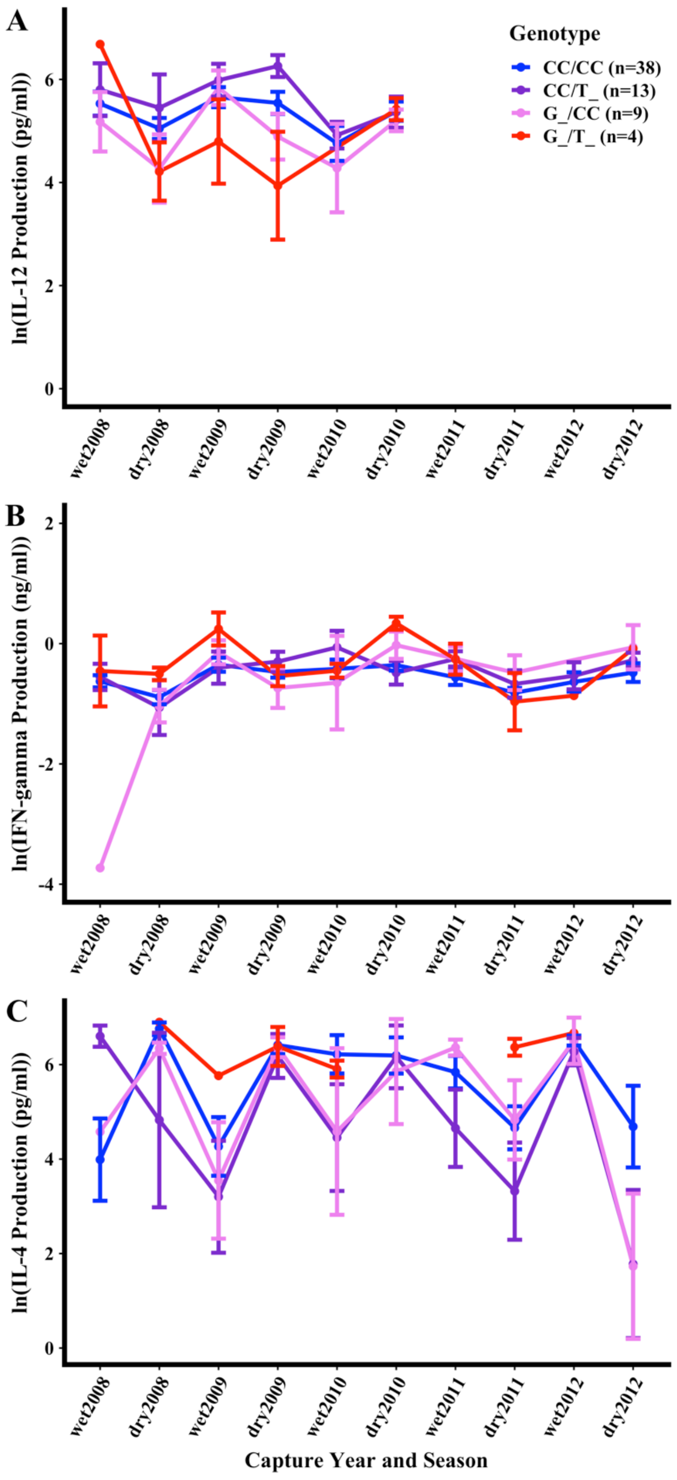

Supplemental Figure S2: Cytokine production over time by multi-locus genotype. Animals heterozygous or homozygous for the 'G' risk allele (red or violet) produce 45% less interleukin 12 (IL-12) than CC animals following pokeweed mitogen stimulation of whole blood (top panel). IL-12 production was not significantly associated with variation at SNP3195. Additionally, we observed no detectable difference in interferon gamma (IFNγ) or interleukin 4 (IL-4) production relative to SNP2253 and SNP3195 genotype in this herd.
